# Use of non-prescription analgesic medications and survival among Black women with ovarian cancer

**DOI:** 10.1038/s41416-025-03254-4

**Published:** 2025-11-05

**Authors:** Christelle Colin-Leitzinger, Olga Aranzabal, Courtney E. Johnson, Anthony J. Alberg, Elisa V. Bandera, Melissa Bondy, Michele L. Cote, Theresa A. Hastert, Kristin Haller, Andrew Lawson, Jeffrey R. Marks, Edward S. Peters, Paul D. Terry, Joellen M. Schildkraut, Lauren C. Peres

**Affiliations:** 1 Department of Cancer Epidemiology, Moffitt Cancer Center, Tampa, FL USA; 2 Department of Epidemiology, Emory University, Rollins School of Public Health, Atlanta, GA USA; 3 Department of Epidemiology and Biostatistics, University of South Carolina, Arnold School of Public Health, Columbia, SC USA; 4 Cancer Epidemiology and Health Outcomes, Rutgers Cancer Institute, New Brunswick, NJ USA; 5 Department of Epidemiology and Population Health, Stanford University School of Medicine, Palo Alto, CA USA; 6 Melvin and Bren Simon Comprehensive Cancer Center, Indiana University, Indianapolis, IN USA; 7 Department of Oncology, Wayne State University, Detroit, MI USA; 8 Population Studies and Disparities Research Program, Karmanos Cancer Institute, Detroit, MI USA; 9 Department of Public Health Sciences, College of Medicine, Medical University of South Carolina, Charleston, SC USA; 10 Usher Institute, School of Medicine, University of Edinburgh, Edinburgh, Scotland; 11 Department of Surgery, Duke University School of Medicine, Durham, NC USA; 12 Department of Epidemiology, University of Nebraska Medical Center, Omaha, NE USA; 13 Department of Medicine, University of Tennessee Medical Center, Knoxville, TN USA

**Keywords:** Ovarian cancer, Cancer epidemiology

## Abstract

**Background:**

Chronic inflammation and inflammatory-related exposures have been implicated in epithelial ovarian cancer (EOC) prognosis. However, no studies have evaluated whether analgesic medication use impacts survival in Black women with EOC, an understudied population with poor survival.

**Methods:**

Leveraging data from the African American Cancer Epidemiology Study, we examined the association of pre-diagnostic analgesic medication use (aspirin, non-aspirin non-steroidal anti-inflammatory drugs [naNSAIDs], and acetaminophen) with survival among self-identified Black women diagnosed with EOC (*N* = 541) using multivariable Cox proportional hazards regression. Stratified analyses were conducted by comorbidities and histotype.

**Results:**

Acetaminophen use was associated with a higher risk of mortality overall (HR = 1.40; 95% CI = 1.00–1.97) and for frequent and chronic use (≥30 days per month: HR = 1.62; 95% CI = 1.12–2.34; >5 years: HR = 1.57; 95% CI = 1.03–2.39). These associations were more pronounced among women with high-grade serous carcinoma (HGSC)/carcinosarcoma and those with comorbidities. Among women with comorbidities, naNSAID use was associated with a decreased risk of mortality (HR = 0.71; 95% CI = 0.51–0.99), but no association was observed among women without comorbidities (HR = 0.99; 95% CI = 0.56–1.75). No associations with survival were observed for aspirin.

**Conclusion:**

Chronic use of acetaminophen negatively impacted survival among Black women with EOC, while naNSAID use conferred a survival advantage only among women with comorbidities.

## Introduction

Some analgesic medications, or pain relievers, represent potential approaches for cancer prevention or improving cancer outcomes due to their anti-inflammatory properties. The two most commonly used non-prescription analgesic medications are acetaminophen and non-steroidal anti-inflammatory drugs (NSAIDs). Acetaminophen has weak anti-inflammatory activity and primarily relieves pain by blocking pain signals in the central nervous system [1, 2], whereas NSAIDs relieve pain by reducing inflammation through the inhibition of cyclooxygenase enzymes and subsequent decreases in prostaglandin production [3]. Aspirin is a type of NSAID that exhibits anti-thrombotic properties in addition to its anti-inflammatory effects, resulting in its use as both an analgesic medication and in the prevention of cardiovascular disease [4]. However, adverse effects can occur with chronic use of these medications, namely gastrointestinal bleeding for NSAIDs and liver damage for acetaminophen [5, 6].

Epithelial ovarian cancer (EOC) is one of the deadliest gynaecologic malignancies in the U.S., with a 5-year survival rate of only 51% [7]. Chronic inflammation has been shown to impact the prognosis of EOC as evidenced by associations of inflammatory-related exposures (e.g. obesity, pro-inflammatory diet, physical inactivity, cigarette smoking) and circulating biomarkers with survival [8–12]. Several studies have investigated the association of analgesic medication use with survival in EOC, but findings have been equivocal for NSAIDs. Most studies [13–18] either find no association with mortality for NSAIDs, including aspirin, or a weak inverse association depending on exposure classification and timing of exposure relative to cancer diagnosis. For example, Merritt et al. [18] showed that post-diagnosis rather than pre-diagnosis regular use of aspirin and non-aspirin NSAIDs (naNSAIDs) was associated with better EOC-specific survival in the Nurses’ Health Studies [18]. For acetaminophen, most studies show no association with survival for both pre- and post-diagnostic use [13, 15, 18–20].

Prior evidence on the association of analgesic medications and survival in EOC is from populations of predominantly White women. Black women with EOC experience lower survival rates compared to White women [7]. Yet, Black women are often underrepresented in ovarian cancer research, and to date, no studies have examined the role of analgesic medications in outcomes of Black women diagnosed with EOC. The present study investigates the relationship between pre-diagnostic, non-prescription analgesic medication use and survival among Black women with EOC.

## Methods

### Study population

We utilised data from the African American Cancer Epidemiology Study (AACES) [21], a population-based study of Black or African American women diagnosed with EOC. Study participants were identified through rapid case ascertainment at SEER or state cancer registries, gynaecologic oncology departments and hospitals. Eligible participants self-identified as Black or African American, were aged 20–79 years, were diagnosed with invasive EOC between December 2010 and December 2015, and resided in 11 geographic regions across the U.S.: Alabama, Georgia, Illinois, Louisiana, Michigan [Detroit], New Jersey, North Carolina, Ohio, South Carolina, Tennessee and Texas. A baseline telephone interview was completed to obtain information on demographics, reproductive history, medication use and lifestyle activities (e.g. smoking, physical activity). A shortened questionnaire was available for women who might otherwise have refused. Institutional Review Board approval was obtained at each site, and informed consent was obtained from all participants.

### Analgesic medication use

Participants were asked to recall any pain or inflammation medications they had taken regularly (at least once a week or at least 5 days per month) at any point in their lives. To aid in recollection, examples of these medications and their uses were provided. Participants who reported ever using such medications were asked to report the medication name, reason for use, age at first and last use and frequency and duration of use. This process was repeated for each medication used. The medications were then categorised into three groups: aspirin, naNSAIDs and acetaminophen. Combination medications (e.g. Excedrin includes aspirin and acetaminophen) were categorised as both medication types. To determine pre-diagnostic medication use, we categorised women who initiated analgesic medication use within the year before diagnosis or any time after diagnosis as non-users. We also considered women who reported a duration of use less than 6 months as non-users in order to capture the regular users of these medications. Since the short version of the questionnaire did not enquire about analgesic medication use, we only included participants who completed the long version of the questionnaire. Post-diagnostic analgesic medication use was not systematically collected in AACES and was not examined in this analysis.

### Statistical analysis

Participant characteristics were summarised using descriptive statistics and compared by analgesic medication use using the Wilcoxon rank sum or chi-square tests. Vital status was determined using data from the National Death Index, cancer registries, the LexisNexis database and patient contact. Follow-up time was calculated as the time from the interview to death or the date of last contact. Multivariable Cox proportional hazard (PH) models were used to estimate hazard ratios (HR) and 95% confidence intervals (CI) for the association of analgesic medication use (ever, frequency [<30 and ≥30 days/month], and duration [≤5 years, >5 years]) with risk of all-cause mortality. The categories for frequency and duration of use were determined by prior literature and data distribution. Several potential confounders were considered in multivariable models, including age (continuous, years), geographic region (North [Illinois, Michigan, New Jersey and Ohio], Southeast [Georgia, North Carolina, South Carolina and Tennessee], and Southwest [Alabama, Texas, Louisiana]), stage (I, II, III and IV), histotype (high-grade serous carcinoma [HGSC]/carcinosarcoma, not HGSC/carcinosarcoma), smoking status (ever, never), physical activity (met vs. did not meet Physical Activity Guidelines for Americans [22]), body mass index ([BMI]; continuous, kg/m^2^), Charlson comorbidity index [23] ([CCI]; 0, ≥1), education (high school graduate/GED or less, some college or college graduate), income (<$25,000, $25,000 to $74,999, ≥$75,000), insurance (any private insurance, any Medicaid and any Medicare as three separate variables) and debulking status (optimal, suboptimal). Of these, smoking status, physical activity, education and income did not appreciably change the results and were not included in the final models. Additionally, the use of each analgesic medication was included in the models to determine the independent effect of each analgesic medication, adjusting for the use of the others. Adjusted Kaplan-Meier curves were generated using the ggadjustedcurves() function in the *survminer* R package. The PH assumption was tested by evaluating Schoenfeld residuals and time x covariate interactions individually and collectively across all covariates. Histotype violated the PH assumption (*p* < 0.05) and was included as a strata term in the models. Stratified analyses were performed by histotype and the CCI (0 vs. ≥1).

Due to missing data on key covariates included in the models (ranging from 0.3% for insurance and 34% for debulking status), we imputed missing data using the multiple imputation by chained equations (‘mice’) R package. A Cox PH model was estimated, including the analgesic medications and all potential confounders described above, as well as the Nelson–Aalen estimate of the cumulative hazard [24]. The fraction of missing information was used to set the number of imputations (*n* = 39). The imputed and observed distributions of variables with missing data were comparable (Supplementary Table 1). As a sensitivity analysis, the primary analyses were repeated, restricting to participants with complete data.

## Results

A total of 541 Black women enrolled in AACES completed the long version of the baseline questionnaire. The median age at diagnosis was 57 years, and most women were diagnosed with late-stage disease (66%) and HGSC/carcinosarcoma (71%; Table 1). The prevalence of medication use was highest for naNSAIDs (22%), followed by aspirin (17%) and acetaminophen (11%). Among analgesic users, most exhibited daily use, as indicated by a frequency of ≥30 days per month (82% for aspirin, 77% for naNSAIDs, 82% for acetaminophen), and used analgesics for >5 years (55% for aspirin, 58% naNSAIDs, 65% acetaminophen). While aspirin and naNSAID users were less likely to report concurrent use of other analgesic medications, more than half of acetaminophen users also used the other types of analgesic medications (Supplementary Fig. 1). Only eight women reported using all three types of analgesic medications. For each analgesic medication, ever users had a higher BMI and CCI compared with never users (Supplementary Table 2). Additionally, ever users of aspirin were older and more likely to be diagnosed with HGSC/carcinosarcoma and have Medicare insurance compared with never users. A higher proportion of naNSAID users were diagnosed with earlier-stage disease, and fewer had HGSC/carcinosarcoma and Medicaid insurance.

**Table 1:** Participant characteristics.

Characteristics	*N* = 541
Age at diagnosis (years), Median (Range)	57 (20, 79)
Stage, *n* (%)	
I	118 (23%)
II	51 (10%)
III	301 (59%)
IV	37 (7.3%)
Unknown	34
Histotype, *n* (%)	
Non-HGSC/carcinosarcoma	157 (29%)
HGSC/carcinosarcoma	378 (71%)
Unknown	6
BMI (kg/m^2^), Median (Range)	31 (15, 74)
<25, *n* (%)	81 (15%)
25–30, *n* (%)	143 (27%)
≥30, *n* (%)	314 (58%)
Unknown	3
Smoking status, *n* (%)	
Never	299 (55%)
Ever	242 (45%)
Met physical activity guidelines for Americans, *n* (%)	
No	393 (75%)
Yes	130 (25%)
Unknown	18
Income, *n* (%)	
<$25,000	240 (45%)
$25,000–$74,999	211 (40%)
≥$75,000	80 (15%)
Unknown	10
Private insurance, *n* (%)	
No	325 (60%)
Yes	214 (40%)
Unknown	2
Medicare insurance, *n* (%)	
No	391 (73%)
Yes	148 (27%)
Unknown	2
Medicaid insurance, *n* (%)	
No	417 (77%)
Yes	122 (23%)
Unknown	2
Education, *n* (%)	
High school graduate/GED or less	276 (51%)
Some college or college graduate	265 (49%)
CCI, *n* (%)	
0	225 (42%)
1	125 (23%)
≥2	182 (34%)
Unknown	9
Debulking status, *n* (%)	
Suboptimal	109 (31%)
Optimal	246 (69%)
Unknown	186

*HGSC* high-grade serous carcinoma, *BMI* body mass index, *GED* General Educational Development, *CCI* Charlson comorbidity index.

**Fig. 1:**
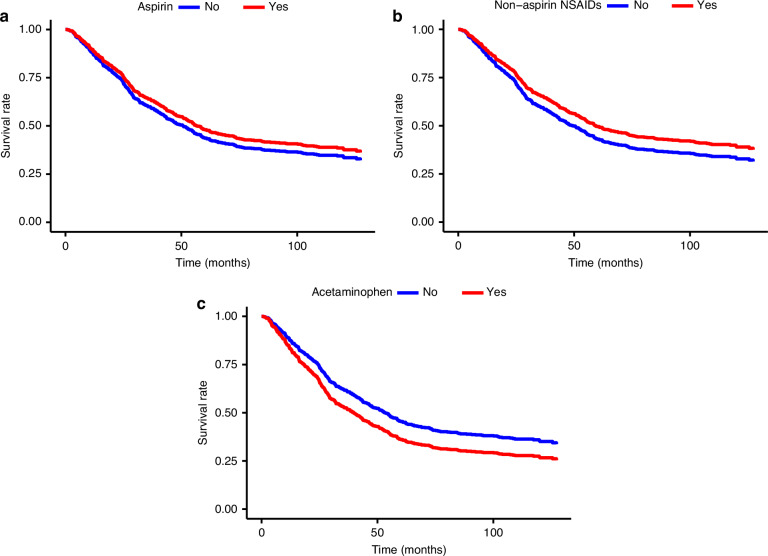
Ever use of analgesic medications and survival. Adjusted Kaplan-Meier survival curves for the association of ever use of **a** aspirin, **b** non-aspirin NSAIDs and **c** acetaminophen with risk of all-cause mortality. Models were adjusted for the other analgesic medications, age, study site, stage, CCI, BMI, insurance and debulking status, and histotype was included as a strata term due to violations of proportional hazards. NSAID non-steroidal anti-inflammatory drug, CCI Charlson Comorbidity Index, BMI body mass index.

During a median follow-up of 4.3 years (range, 0.1–12.2 years), 354 deaths (65%) occurred. For both aspirin and naNSAIDs, an inverse but imprecise association was observed with survival (Table 2, Fig. 1), which persisted when examining frequency and duration of use. A higher risk of mortality was observed with ever use of acetaminophen (HR = 1.40; 95% CI = 1.00–1.97), which was driven by more frequent or daily users (≥30 days per month, HR = 1.62; 95% CI = 1.12–2.34) and a longer duration of use (>5 years, HR = 1.57; 95% CI = 1.03–2.39). Our findings remained consistent in the complete case analysis (Supplementary Table 3).

**Table 2:** Hazard ratios and 95% confidence intervals for the association of analgesic medication use with risk of all-cause mortality overall and stratified by histotype (HGSC/carcinosarcoma vs. non-HGSC/carcinosarcoma) and CCI score (CCI of 0 vs. CCI ≥ 1).

			Histotype^b^				CCI^c^			
	Overall^a^		HGSC/carcinosarcoma		Non-HGSC/carcinosarcoma^d^		CCI of 0		CCI ≥ 1	
Analgesic medication	*N* (Event *N*)	HR (95% CI)	*N* (Event *N*)	HR (95% CI)	*N* (Event *N*)	HR (95% CI)	*N* (Event *N*)	HR (95% CI)	*N* (Event *N*)	HR (95% CI)
Aspirin										
Never	449 (286)	1.00 (Reference)	308 (224)	1.00 (Reference)	141 (62)	1.00 (Reference)	209 (126)	1.00 (Reference)	240 (160)	1.00 (Reference)
Ever	92 (68)	0.86 (0.64, 1.15)	73 (58)	0.81 (0.58, 1.13)	19 (10)	0.97 (0.45, 2.11)	19 (14)	0.97 (0.52, 1.81)	73 (54)	0.85 (0.60, 1.19)
Frequency										
<30	17 (10)	0.70 (0.37, 1.34)	13 (9)	0.66 (0.33, 1.30)	--	--	5 (2)	0.47 (0.11, 2.03)	12 (8)	0.79 (0.38, 1.65)
≥30	75 (58)	0.90 (0.65, 1.24)	60 (49)	0.88 (0.61, 1.27)	--	--	14 (12)	1.31 (0.66, 2.62)	61 (46)	0.86 (0.59, 1.24)
Duration										
≤5 years	41 (29)	0.81 (0.54, 1.21)	31 (24)	0.75 (0.47, 1.17)	10 (5)	1.02 (0.38, 2.79)	12 (9)	1.12 (0.53, 2.37)	29 (20)	0.72 (0.44, 1.18)
>5 years	51 (39)	0.89 (0.61, 1.30)	42 (34)	0.88 (0.57, 1.34)	9 (5)	0.78 (0.28, 2.21)	7 (5)	0.86 (0.30, 2.45)	44 (34)	0.96 (0.63, 1.44)
Non-aspirin NSAIDs										
Never	422 (286)	1.00 (Reference)	308 (234)	1.00 (Reference)	114 (52)	1.00 (Reference)	197 (123)	1.00 (Reference)	225 (163)	1.00 (Reference)
Ever	119 (68)	0.79 (0.60, 1.05)	73 (48)	0.76 (0.55, 1.06)	46 (20)	0.92 (0.50, 1.68)	31 (17)	0.99 (0.56, 1.75)	88 (51)	0.71 (0.51, 0.99)
Frequency										
<30	27 (16)	0.74 (0.44, 1.25)	19 (11)	0.59 (0.31, 1.11)	--	--	7 (4)	0.57 (0.20, 1.65)	20 (12)	0.82 (0.44, 1.51)
≥30	92 (52)	0.78 (0.57, 1.06)	54 (37)	0.81 (0.56, 1.16)	--	--	24 (13)	1.25 (0.65, 2.40)	68 (39)	0.67 (0.46, 0.97)
Duration										
≤5 years	50 (30)	0.87 (0.59, 1.28)	34 (21)	0.74 (0.46, 1.17)	16 (9)	1.49 (0.60, 3.71)	12 (7)	0.87 (0.39, 1.98)	38 (23)	0.83 (0.53, 1.31)
>5 years	69 (38)	0.72 (0.50, 1.03)	39 (27)	0.76 (0.50, 1.17)	30 (11)	0.57 (0.27, 1.20)	19 (10)	1.19 (0.55, 2.56)	50 (28)	0.61 (0.40, 0.93)
Acetaminophen										
Never	484 (312)	1.00 (Reference)	342 (249)	1.00 (Reference)	142 (63)	1.00 (Reference)	213 (129)	1.00 (Reference)	271 (183)	1.00 (Reference)
Ever	57 (42)	1.40 (1.00, 1.97)	39 (33)	1.66 (1.12, 2.44)	18 (9)	1.29 (0.56, 2.99)	15 (11)	1.13 (0.57, 2.21)	42 (31)	1.46 (0.97, 2.19)
Frequency										
<30	10 (6)	0.73 (0.32, 1.70)	6 (6)	1.56 (0.65, 3.72)	--	--	4 (2)	1.36 (0.31, 6.00)	6 (4)	0.75 (0.27, 2.11)
≥30	47 (36)	1.62 (1.12, 2.34)	33 (27)	1.63 (1.07, 2.50)	--	--	11 (9)	0.95 (0.43, 2.09)	36 (27)	1.69 (1.09, 2.61)
Duration										
≤5 years	20 (14)	1.14 (0.65, 1.99)	13 (12)	1.73 (0.94, 3.21)	7 (2)	0.37 (0.08, 1.77)	6 (4)	1.71 (0.59, 4.93)	14 (10)	1.21 (0.63, 2.35)
>5 years	37 (28)	1.57 (1.03, 2.39)	26 (21)	1.56 (0.95, 2.56)	11 (7)	2.45 (0.94, 6.36)	9 (7)	0.92 (0.35, 2.40)	28 (21)	1.54 (0.94, 2.51)

*HGSC* high-grade serous carcinoma, *CCI* Charlson comorbidity index, *HR* hazard ratio, *CI* confidence interval, *NSAID* non-steroidal anti-inflammatory drug, *BMI* body mass index.

^a^Models adjusted for the other analgesic medications, age, study site, stage, CCI, BMI, insurance and debulking status. Histotype is included as a strata term due to violations of proportional hazards.

^b^Models were adjusted for the other analgesic medications, age, study site, stage, CCI, BMI, insurance and debulking status.

^c^Models were adjusted for the other analgesic medications, age, study site, stage, BMI, insurance and debulking status. Histotype is included as a strata term due to violations of proportional hazards.

^d^Due to small sample sizes, effect estimates for frequency of use and risk of mortality among women with non-HGSC/carcinosarcoma are not provided.

**Fig. 2:**
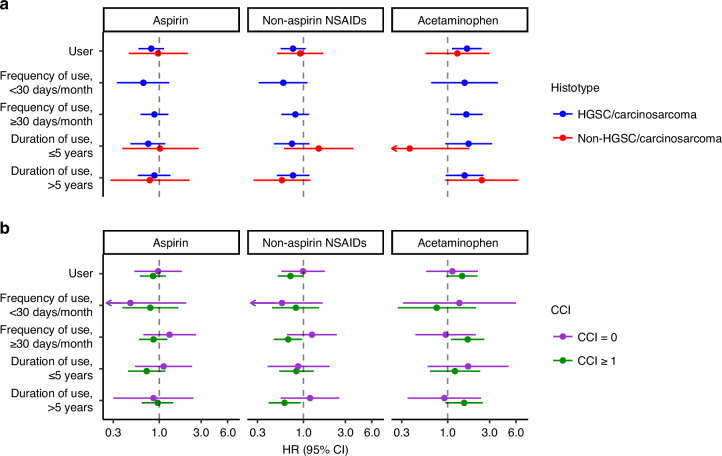
Analgesic medication use and survival by histotype and CCI. Hazard ratios and 95% confidence intervals for the association of analgesic medication use with risk of all-cause mortality stratified by **a** histotype (HGSC/carcinosarcoma vs. non-HGSC/carcinosarcoma) and **b** CCI (CCI of 0 vs. CCI ≥ 1). The histotype models were adjusted for the other analgesic medications, age, study site, stage, CCI, BMI, insurance and debulking status. The CCI models were adjusted for the other analgesic medications, age, study site, stage, BMI, insurance and debulking status, and histotype was included as a strata term due to violations of proportional hazards. HGSC high-grade serous carcinoma, CCI Charlson comorbidity index, BMI body mass index, NSAID non-steroidal anti-inflammatory drug.

Table 2 and Fig. 2 provide the survival associations by histotype and the CCI. When stratifying by histotype, we observed that the increased risk of mortality for acetaminophen use was present specifically among women with HGSC/carcinosarcoma (HR = 1.66; 95% CI = 1.12–2.44), while an elevated risk of mortality was observed for acetaminophen use among women with non-HGSC/carcinosarcoma, although CIs were wide (HR = 1.29; 95% CI = 0.56–2.99). Likewise, a higher risk of mortality for a higher frequency and a longer duration of acetaminophen use was present among HGSC/carcinosarcoma. However, small sample sizes of non-HGSC/carcinosarcoma resulted in imprecise estimates for acetaminophen duration analyses and precluded the investigation of frequency of use. No appreciable differences in the associations of aspirin and naNSAIDs with survival were observed by histotype.

Stratifying by the CCI showed that analgesic medication use was not associated with survival among women without comorbidities (CCI = 0). Among women with comorbidities (CCI ≥ 1), we observed an increased risk of mortality for acetaminophen use (HR = 1.46; 95% CI = 0.97–2.19) and a decreased risk of mortality for naNSAID use (HR = 0.71; 95% CI = 0.51–0.99). These associations were pronounced in magnitude for more frequent use (acetaminophen: HR = 1.69; 95% CI = 1.09–2.61 and naNSAIDS: HR = 0.67; 95% CI = 0.46–0.97, respectively) and longer durations of use (acetaminophen: HR = 1.54; 95% CI = 0.94–2.51 and naNSAIDs: HR = 0.61; 95% CI = 0.40–0.93, respectively). No associations were observed for aspirin use and survival by CCI.

## Discussion

In a cohort of Black women diagnosed with EOC, we observed a higher risk of mortality for pre-diagnostic acetaminophen use, particularly those reporting daily use and/or 5 years or more of use. These associations occurred primarily among women with HGSC/carcinosarcoma and those with comorbid conditions. We also observed a lower risk of mortality for naNSAID use overall, although estimates were imprecise, and among women with comorbid conditions. However, no associations with survival were observed for aspirin use.

Despite observing worse survival for acetaminophen use in the present study, most existing research suggests that pre-diagnostic acetaminophen use is not associated with ovarian cancer survival [13–15, 18, 20]. The conflicting findings between our study and prior literature may be due to inherent differences in the attributes of the study populations. Our cohort is comprised of Black women with EOC residing in the U.S., while prior studies included populations of mostly White women with EOC residing in the U.S. or Australia[13–15, 18, 20]. We also found that acetaminophen users in the present study were more likely to report taking acetaminophen at a high frequency and for long durations compared with acetaminophen users in prior literature. Women who used acetaminophen daily or for more than 5 years prior to diagnosis had the highest risk of mortality in our study, suggesting that the association of pre-diagnostic acetaminophen use with survival was largely driven by more frequent or chronic use. These findings may be attributed to the way acetaminophen is processed and its long-term physiologic effects. Acetaminophen is primarily metabolised in the liver by forming conjugates with glucuronide and sulphate, but up to 10% is oxidised to form a reactive toxic intermediate, N-acetyl-p-benzoquinone imine (NAPQI) [25, 26]. Chronic acetaminophen use can lead to an increased production of NAPQI, causing oxidative stress and damage to liver cells, potentially leading to liver dysfunction or failure [25, 26]. These effects may differ by race, as studies have shown that while Black/African populations have a greater clearance of acetaminophen compared to White populations [27], Black/African individuals metabolise acetaminophen by oxidation at a slower rate [28, 29]. However, more work is needed to better understand acetaminophen pharmacokinetics in diverse populations. Additionally, chemotherapy dose adjustments may be applied to prevent further toxicity for patients with liver dysfunction [30], and in ovarian cancer, impaired liver function was associated with chemotherapy dose modifications, treatment discontinuation and worse overall survival [31]. Besides liver impacts, acetaminophen and its metabolites are excreted through the urine, and long-term use can strain the kidneys, resulting in renal impairment or failure [5]. Long-term acetaminophen use has also been linked to hypertension and can induce oxidative stress and inflammation, contributing to endothelial dysfunction and atherosclerosis [5]. Additional work is needed to elucidate the biologic pathways underlying the increased risk of mortality associated with chronic acetaminophen use.

We observed inverse associations between pre-diagnostic aspirin and naNSAID use and survival overall, although CIs were wide and imprecise. These findings are similar to most prior studies considering pre-diagnostic use of aspirin or naNSAIDs [13–18]. One study showed that frequent (≥4 days per week) pre-diagnostic NSAID use was associated with better survival in a cohort of Australian women with ovarian cancer [20]. However, comparing findings across studies is challenging due to the lack of standard medication use questionnaires and varying definitions of regular use. For example, Dixon et al. [15] defined regular use as at least once a week, while Merritt et al. [18] defined regular use as ≥2 days per week. Nevertheless, studies consistently show that post-diagnostic NSAID use may be a key factor in ovarian cancer outcomes. In the study of Australian women with ovarian cancer and in the Nurses’ Health Studies [18, 20], post-diagnostic use of naNSAIDs and aspirin was associated with better survival. The present study was unable to reliably assess post-diagnostic use of analgesic medications, but this is likely to be a fruitful avenue for future research.

For both acetaminophen and naNSAIDs, heterogeneity was observed in the survival associations by comorbidities. Among women with comorbidities, we found a higher risk of mortality for acetaminophen use but a lower risk of mortality for naNSAIDs. Conversely, analgesic medication use was not associated with survival among women without comorbidities. These findings may be due to differences in patterns of analgesic medication use by comorbidity status. In our study population, women with comorbidities were more likely to report analgesic medication use as well as a longer duration of use than women without comorbidities. Additionally, women with comorbidities may use certain types of analgesic medications more than others due to potential contraindications [32]. For example, individuals with gastrointestinal diseases are advised to use acetaminophen due to the known risks of gastrointestinal bleeding with continued NSAID use. Drug interactions can also occur between analgesics and medications taken for comorbid conditions, resulting in side effects that could have downstream impacts on morbidity and mortality among women with comorbidities (e.g. concurrent anticoagulant or antidepressant use with NSAIDs increases risk of gastrointestinal bleeding, concurrent use of antidiabetic agents and aspirin increases risk of hypoglycemic effects) [33]. Lastly, our findings may be compounded by the higher prevalence of some comorbidities that are indications for analgesic medications among Black individuals compared to White individuals (e.g. cardiovascular disease) [34].

The histotype stratified analyses did not provide clear evidence to suggest there was heterogeneity in the survival associations by histotype. We observed that the higher risk of mortality for acetaminophen use was present specifically among women with HGSC/carcinosarcoma, with an attenuated association observed among women with non-HGSC/carcinosarcoma, although CIs were wide. The rarity of non-HGSC/carcinosarcoma precluded our ability to investigate frequency of use, limited our power to detect associations for duration of use, and did not allow for stratified analyses within each individual histotype (e.g. endometrioid, clear cell). Thus, these findings should be interpreted with caution and would benefit from further investigation in a larger cohort.

The present study possesses several notable strengths. It is the only study to exclusively focus on analgesic use and ovarian cancer survival among Black women, leveraging data from a well-established population-based study. The AACES features a relatively large cohort of Black women in the U.S. and includes detailed information on analgesic medication use, allowing for effect estimates based on frequency and duration of use, and the ability to adjust for relevant confounders. However, this work also has limitations. The data on analgesic medication use were obtained through self-report, which may be impacted by recall bias, particularly with respect to frequency and duration of use. Moreover, the variety of analgesic medications that could be used over the lifetime and for different indications poses additional recall challenges. Medication use collected from registers or medical records may be more accurate, particularly in the setting of prescription medications, but could suffer from exposure misclassification as over-the-counter medications are not always captured and information on medication compliance is typically unavailable. We were not able to investigate the effect of concurrent analgesic medication use and recency of use due to small stratum sample sizes or to evaluate medication dosing, as study participants did not consistently report this information. While several confounders were considered in our analyses, there is still a possibility of bias due to residual or unmeasured confounding. Although AACES included Black women from 11 sites in the U.S., our findings may not be generalisable to all Black women in the U.S., particularly in the Western region of the country, or other racial and ethnic groups. Additionally, despite having missing data on key covariates, we used multiple imputation and observed consistent findings when restricting analyses to participants with complete data. Lastly, we do not have data on recurrence or cause of death in the entire cohort, precluding examinations of progression-free or cause-specific survival.

In summary, we observed that chronic and frequent acetaminophen use negatively impacted survival among Black women with ovarian cancer, particularly among women with comorbidities, while overall and chronic naNSAID use was associated with better survival only among women with comorbidities. These findings provide valuable insights into potential factors affecting survival among Black women diagnosed with EOC, an understudied population in ovarian cancer research. The evidence base on this topic will be strengthened by investigating the impact of both pre- and post-diagnostic analgesic medication use on survival in larger, more diverse cohorts. Moreover, our lack of understanding of the mechanisms and pathways through which analgesic medications influence survival in cancer patients also warrants further investigation.

## Supplementary information

Supplementary Material

## Data Availability

Data from this study are available from the corresponding author on reasonable request and approval by the study investigators.
