# Supplementary material for: Use of non-prescription analgesic medications and survival among Black women with ovarian cancer
Source: Br J Cancer. 2025 Nov 5;134(2):237–44. doi: 10.1038/s41416-025-03254-4 (PMC12820352; doi:10.1038/s41416-025-03254-4)

**SUPPLEMENTARY MATERIAL**

**Supplementary Table 1.** Comparison of observed and imputed values of imputed covariates

| **Characteristic** | **Observed** | **Imputed** |
| --- | --- | --- |
| **Stage** |  |  |
| I | 118 (23%) | 125 (23%) |
| II | 51 (10%) | 55 (10%) |
| III | 301 (59%) | 318 (59%) |
| IV | 37 (7.3%) | 43 (7.9%) |
| Unknown | 34 | 0 |
| **CCI** |  |  |
| 0 | 225 (42%) | 228 (42%) |
| 1 | 125 (24%) | 128 (24%) |
| ≥ 2 | 182 (34%) | 185 (34%) |
| Unknown | 9 | 0 |
| **BMI, kg/m^2^** |  |  |
| <25 | 81 (15%) | 82 (15%) |
| 25-30 | 143 (27%) | 143 (26%) |
| ≥30 | 314 (58%) | 316 (58%) |
| Unknown | 3 | 0 |
| **Private insurance** |  |  |
| No | 325 (60%) | 326 (60%) |
| Yes | 214 (40%) | 215 (40%) |
| Unknown | 2 | 0 |
| **Medicare insurance** |  |  |
| No | 391 (73%) | 392 (72%) |
| Yes | 148 (27%) | 149 (28%) |
| Unknown | 2 | 0 |
| **Medicaid insurance** |  |  |
| No | 417 (77%) | 418 (77%) |
| Yes | 122 (23%) | 123 (23%) |
| Unknown | 2 | 0 |
| **Met physical activity guidelines for Americans** |  |  |
| No | 393 (75%) | 403 (74%) |
| Yes | 130 (25%) | 138 (26%) |
| Unknown | 18 | 0 |
| **Income** |  |  |
| < $25,000 | 240 (45%) | 245 (45%) |
| $25,000-$74,999 | 211 (40%) | 215 (40%) |
| ≥ $75,000 | 80 (15%) | 81 (15%) |
| Unknown | 10 | 0 |
| **Debulking status** |  |  |
| Suboptimal | 109 (31%) | 166 (31%) |
| Optimal | 246 (69%) | 375 (69%) |
| Unknown | 186 | 0 |

**Supplementary Table 2.** Participant characteristics by use of analgesic medications

|  | **Aspirin Use (N = 541)** | | | **Non-aspirin NSAID Use (N = 541)** | | | | **Acetaminophen Use (N = 541)** | | | |
| --- | --- | --- | --- | --- | --- | --- | --- | --- | --- | --- | --- |
| **Characteristics** | **No**, N = 449 | **Yes**, N = 92 | **p-value** | | **No**, N = 422 | **Yes**, N = 119 | **p-value** | | **No**, N = 484 | **Yes**, N = 57 | **p-value** |
| **Age at diagnosis (years), Median (Range)** | 56 (20, 79) | 64 (35, 78) | <0.001 | | 57 (20, 79) | 58 (20, 79) | 0.9 | | 57 (20, 79) | 61 (33, 79) | 0.4 |
| **Stage, n (%)** |  |  | 0.4 | |  |  | 0.047 | |  |  | 0.08 |
| I | 101 (24%) | 17 (20%) |  | | 83 (21%) | 35 (32%) |  | | 109 (24%) | 9 (17%) |  |
| II | 43 (10%) | 8 (9.2%) |  | | 38 (9.6%) | 13 (12%) |  | | 41 (9.1%) | 10 (19%) |  |
| III | 249 (59%) | 52 (60%) |  | | 248 (62%) | 53 (48%) |  | | 272 (60%) | 29 (54%) |  |
| IV | 27 (6.4%) | 10 (11%) |  | | 28 (7.1%) | 9 (8.2%) |  | | 31 (6.8%) | 6 (11%) |  |
| Unknown | 29 | 5 |  | | 25 | 9 |  | | 31 | 3 |  |
| **Histotype, n (%)** |  |  | 0.03 | |  |  | 0.03 | |  |  | 0.9 |
| Non HGSC/carcinosarcoma | 139 (31%) | 18 (20%) |  | | 113 (27%) | 44 (38%) |  | | 140 (29%) | 17 (30%) |  |
| HGSC/carcinosarcoma | 305 (69%) | 73 (80%) |  | | 305 (73%) | 73 (62%) |  | | 339 (71%) | 39 (70%) |  |
| Unknown | 5 | 1 |  | | 4 | 2 |  | | 5 | 1 |  |
| **BMI (kg/m^2^), Median (Range)** | 31 (15, 74) | 33 (21, 61) | 0.03 | | 31 (15, 74) | 33 (17, 65) | 0.02 | | 31 (15, 74) | 33 (17, 65) | 0.02 |
| Unknown | 3 | 0 |  | | 3 | 0 |  | | 3 | 0 |  |
| **BMI categories, n (%)** |  |  | 0.02 | |  |  | 0.08 | |  |  | 0.3 |
| <25 kg/m² | 76 (17%) | 5 (5.4%) |  | | 68 (16%) | 13 (11%) |  | | 76 (16%) | 5 (8.8%) |  |
| 25-30 kg/m² | 118 (26%) | 25 (27%) |  | | 117 (28%) | 26 (22%) |  | | 129 (27%) | 14 (25%) |  |
| ≥30 kg/m² | 252 (57%) | 62 (67%) |  | | 234 (56%) | 80 (67%) |  | | 276 (57%) | 38 (67%) |  |
| Unknown | 3 | 0 |  | | 3 | 0 |  | | 3 | 0 |  |
| **Smoking status, n (%)** |  |  | 0.07 | |  |  | 0.4 | |  |  | 0.9 |
| Never | 256 (57%) | 43 (47%) |  | | 237 (56%) | 62 (52%) |  | | 268 (55%) | 31 (54%) |  |
| Ever | 193 (43%) | 49 (53%) |  | | 185 (44%) | 57 (48%) |  | | 216 (45%) | 26 (46%) |  |
| **Met physical activity guidelines, n (%)** | 104 (24%) | 26 (30%) | 0.3 | | 106 (26%) | 24 (21%) | 0.3 | | 122 (26%) | 8 (14%) | 0.05 |
| Unknown | 14 | 4 |  | | 11 | 7 |  | | 17 | 1 |  |
| **Income, n (%)** |  |  | 0.5 | |  |  | >0.9 | |  |  | 0.9 |
| < $25,000 | 199 (45%) | 41 (46%) |  | | 189 (45%) | 51 (44%) |  | | 213 (45%) | 27 (47%) |  |
| $25,000-$74,999 | 179 (41%) | 32 (36%) |  | | 164 (39%) | 47 (41%) |  | | 190 (40%) | 21 (37%) |  |
| ≥ $75,000 | 63 (14%) | 17 (19%) |  | | 63 (15%) | 17 (15%) |  | | 71 (15%) | 9 (16%) |  |
| Unknown | 8 | 2 |  | | 6 | 4 |  | | 10 | 0 |  |
| **Private insurance, n (%)** |  |  | 0.1 | |  |  | 0.6 | |  |  | 0.1 |
| No | 264 (59%) | 61 (68%) |  | | 256 (61%) | 69 (58%) |  | | 286 (59%) | 39 (70%) |  |
| Yes | 185 (41%) | 29 (32%) |  | | 165 (39%) | 49 (42%) |  | | 197 (41%) | 17 (30%) |  |
| Unknown | 0 | 2 |  | | 1 | 1 |  | | 1 | 1 |  |
| **Medicare insurance, n (%)** |  |  | <0.001 | |  |  | 0.2 | |  |  | 0.3 |
| No | 339 (76%) | 52 (58%) |  | | 311 (74%) | 80 (68%) |  | | 354 (73%) | 37 (66%) |  |
| Yes | 110 (24%) | 38 (42%) |  | | 110 (26%) | 38 (32%) |  | | 129 (27%) | 19 (34%) |  |
| Unknown | 0 | 2 |  | | 1 | 1 |  | | 1 | 1 |  |
| **Medicaid insurance, n (%)** |  |  | 0.4 | |  |  | 0.02 | |  |  | 0.7 |
| No | 344 (77%) | 73 (81%) |  | | 316 (75%) | 101 (86%) |  | | 375 (78%) | 42 (75%) |  |
| Yes | 105 (23%) | 17 (19%) |  | | 105 (25%) | 17 (14%) |  | | 108 (22%) | 14 (25%) |  |
| Unknown | 0 | 2 |  | | 1 | 1 |  | | 1 | 1 |  |
| **Education, n (%)** |  |  | 0.2 | |  |  | 0.9 | |  |  | >0.9 |
| High school graduate/  GED or less | 223 (50%) | 53 (58%) |  | | 216 (51%) | 60 (50%) |  | | 247 (51%) | 29 (51%) |  |
| Some college or college  graduate | 226 (50%) | 39 (42%) |  | | 206 (49%) | 59 (50%) |  | | 237 (49%) | 28 (49%) |  |
| **CCI, n (%)** |  |  | <0.001 | |  |  | <0.001 | |  |  | 0.02 |
| 0 | 207 (47%) | 18 (20%) |  | | 194 (47%) | 31 (26%) |  | | 210 (44%) | 15 (26%) |  |
| 1 | 104 (24%) | 21 (23%) |  | | 101 (24%) | 24 (21%) |  | | 111 (23%) | 14 (25%) |  |
| ≥ 2 | 130 (29%) | 52 (57%) |  | | 120 (29%) | 62 (53%) |  | | 154 (32%) | 28 (49%) |  |
| Unknown | 8 | 1 |  | | 7 | 2 |  | | 9 | 0 |  |
| **Debulking status, n (%)** |  |  | 0.11 | |  |  | 0.2 | |  |  | 0.5 |
| Suboptimal | 85 (29%) | 24 (39%) |  | | 90 (32%) | 19 (25%) |  | | 101 (31%) | 8 (25%) |  |
| Optimal | 209 (71%) | 37 (61%) |  | | 188 (68%) | 58 (75%) |  | | 222 (69%) | 24 (75%) |  |
| Unknown | 155 | 31 |  | | 144 | 42 |  | | 161 | 25 |  |

NSAID: non-steroidal anti-inflammatory drug; HGSC: high-grade serous carcinoma; BMI: body mass index; GED: General Educational Development; CCI: Charlson Comorbidity Index

**Supplementary Table 3.** Hazard ratios and 95% confidence intervals for the association of analgesic medication use with risk of all-cause mortality using a complete case analytic approach (restricting to patients with data on all covariates).

| **Analgesic medication** | **N (Event N)** | **HR (95% CI)** |
| --- | --- | --- |
| **Ever use** |  |  |
| **Aspirin** |  |  |
| Never | 177 (278) | 1.00 (Reference) |
| Ever | 46 (59) | 0.92 (0.63, 1.36) |
| **Non-aspirin NSAID** |  |  |
| Never | 182 (264) | 1.00 (Reference) |
| Ever | 41 (73) | 0.65 (0.45, 0.94) |
| **Acetaminophen** |  |  |
| Never | 198 (305) | 1.00 (Reference) |
| Ever | 25 (32) | 1.42 (0.91, 2.21) |
| **Duration** |  |  |
| **Aspirin** |  |  |
| Never user | 177 (278) | 1.00 (Reference) |
| ≤5 years | 22 (28) | 0.89 (0.55, 1.45) |
| >5 years | 24 (31) | 0.93 (0.56, 1.55) |
| **Non-aspirin NSAID** |  |  |
| Never user | 182 (264) | 1.00 (Reference) |
| ≤5 years | 17 (31) | 0.71 (0.42, 1.19) |
| >5 years | 24 (42) | 0.60 (0.38, 0.96) |
| **Acetaminophen** |  |  |
| Never user | 198 (305) | 1.00 (Reference) |
| ≤5 years | 10 (13) | 1.12 (0.58, 2.18) |
| >5 years | 15 (19) | 1.70 (0.95, 3.03) |
| **Frequency** |  |  |
| **Aspirin** |  |  |
| Never user | 177 (278) | 1.00 (Reference) |
| <30 days | 7 (11) | 0.68 (0.30, 1.50) |
| ≥30 days | 39 (48) | 0.98 (0.64, 1.51) |
| **Non-aspirin NSAID** |  |  |
| Never user | 182 (264) | 1.00 (Reference) |
| <30 days | 11 (18) | 0.64 (0.33, 1.24) |
| ≥30 days | 30 (55) | 0.62 (0.41, 0.94) |
| **Acetaminophen** |  |  |
| Never user | 198 (305) | 1.00 (Reference) |
| <30 days | 5 (7) | 0.87 (0.34, 2.19) |
| ≥30 days | 20 (25) | 1.63 (1.00, 2.67) |

HR: hazard ratio; CI: confidence interval; NSAID: non-steroidal anti-inflammatory drug, CCI: Charlson Comorbidity Index, BMI: body mass index.

Models were adjusted for the other analgesic medications, age, study site, stage, CCI, BMI, insurance and debulking status. Histotype is included as a strata term due to violations of proportional hazards.

**Supplementary Figure 1.** Overlap of non-prescription analgesic medication use.


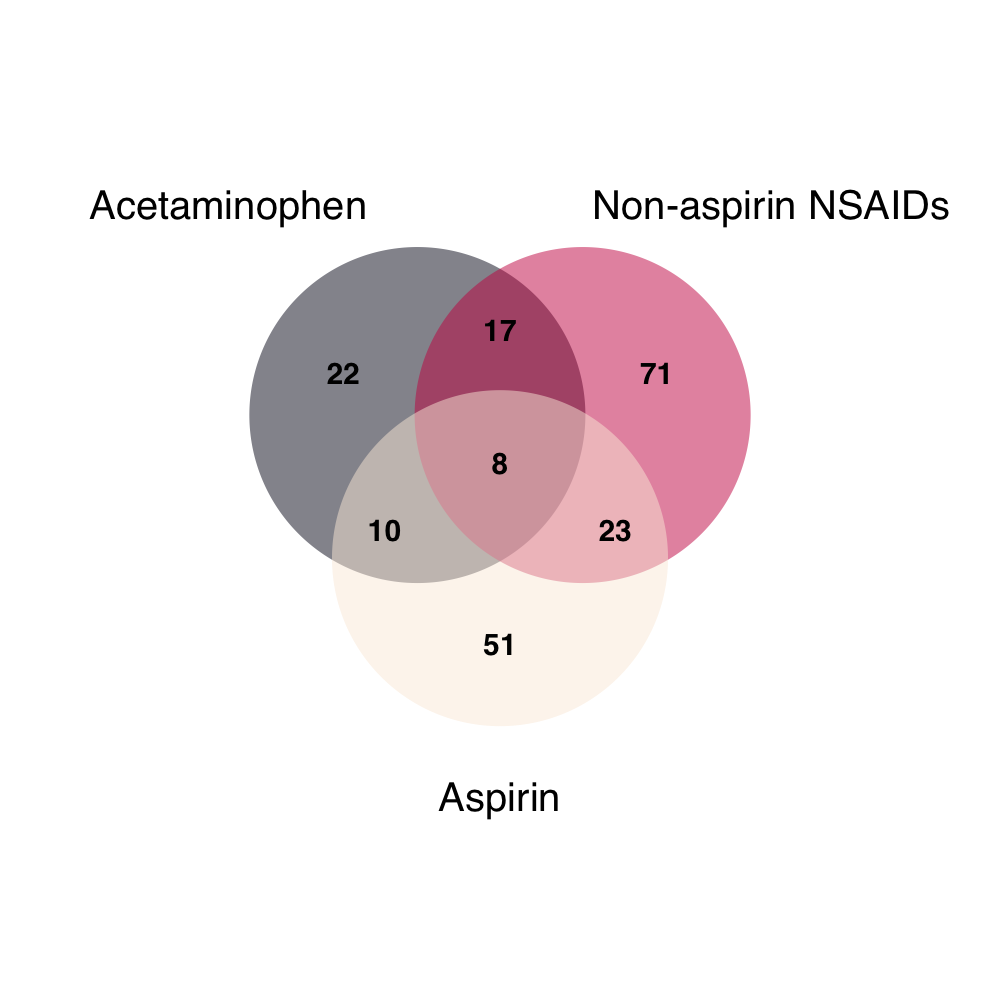

Supplement: Supplementary file 1 — Supplementary Material [file 41416_2025_3254_MOESM1_ESM.docx]
